# Evolution of the T4 phage virion is driven by selection pressure from non-bacterial factors

**DOI:** 10.1128/spectrum.00115-23

**Published:** 2023-09-19

**Authors:** Joanna Majewska, Paulina Miernikiewicz, Aleksander Szymczak, Zuzanna Kaźmierczak, Tomasz M. Goszczyński, Barbara Owczarek, Izabela Rybicka, Jarosław Ciekot, Krystyna Dąbrowska

**Affiliations:** 1 Hirszfeld Institute of Immunology and Experimental Therapy, Polish Academy of Sciences, Wrocław, Poland; 2 Research and Development Center, Regional Specialist Hospital in Wrocław, Wrocław, Poland; University of Exeter, Exeter, United Kingdom

**Keywords:** T4 phage, head proteins, head vertex protein, gp24, gastrointestinal tract, bacteriophage evolution

## Abstract

Bacteriophages colonize animal and human bodies, propagating on sensitive bacteria that are symbionts, commensals, or pathogens of animals and humans. T4-like phages are dependent on abundant symbionts such as *Escherichia coli*, commonly present in animal and human gastrointestinal (GI) tracts. Bacteriophage T4 is one of the most complex viruses, and its intricate structure, particularly the capsid head protecting the phage genome, likely contributes substantially to the overall phage fitness in diverse environments. We investigated how individual head proteins—gp24, Hoc, and Soc—affect T4 phage survival under pressure from non-bacterial factors. We constructed a panel of T4 phage variants defective in these structural proteins: T4∆Soc, T4∆24byp24, T4∆Hoc∆Soc, T4∆Hoc∆24byp24, T4∆Soc∆24byp24, and T4∆Hoc∆Soc∆24byp24 (byp = bypass). These variants were investigated for their sensitivity to selected environmental conditions relevant to the microenvironment of the GI tract, including pH, temperature, and digestive enzymes. The simple and “primitive” structure of the phage capsid (∆24byp24) was significantly less stable at low pH and more sensitive to inactivation by digestive enzymes, and the simultaneous lack of gp24 and Soc resulted in a notable decrease in phage activity at 37°C. Gp24 was also found to be highly resistant to thermal and chemical denaturation. Thus, gp24, which was acquired relatively late in evolution, seems to play a key role in T4 withstanding environmental conditions, including those related to the animal/human GI tract, and Soc is a molecular glue that enhances this protective effect.

**IMPORTANCE:**

Bacteriophages are important components of animal and human microbiota, particularly in the gastrointestinal tract, where they dominate the viral community and contribute to shaping microbial balance. However, interactions with bacterial hosts are not the only element of the equation in phage survival—phages inhabiting the GI tract are constantly exposed to increased temperature, pH fluctuations, or digestive enzymes, which raises the question of whether and how the complex structure of phage capsids contributes to their persistence in the specific microenvironment of human/animal bodies. Here we address this phage-centric perspective, identifying the role of individual head proteins in T4 phage survival in GI tract conditions. The selection pressure driving the evolution of T4-like phages could have come from the external environment that affects phage virions with increased temperature and variable pH; it is possible that in the local microenvironment along the GI tract, the phage benefits from stability-protecting proteins.

## INTRODUCTION

Bacteriophages (phages) colonize human and animal bodies and make up an important part of their microbiota. They are known to populate the gastrointestinal (GI) tract and have been identified as the main component (about 90%) of the gut virome (1). Phage presence is determined by the presence of sensitive bacteria, mostly symbionts such as *Escherichia coli*. T4-like phages are an abundant group of coliphages. Discovered a long time ago and extensively studied over the decades, T4 is also one of the most common model phages in microbiology and biotechnology, being one of the most important representatives of human and animal microbiota.

**Fig 1:**
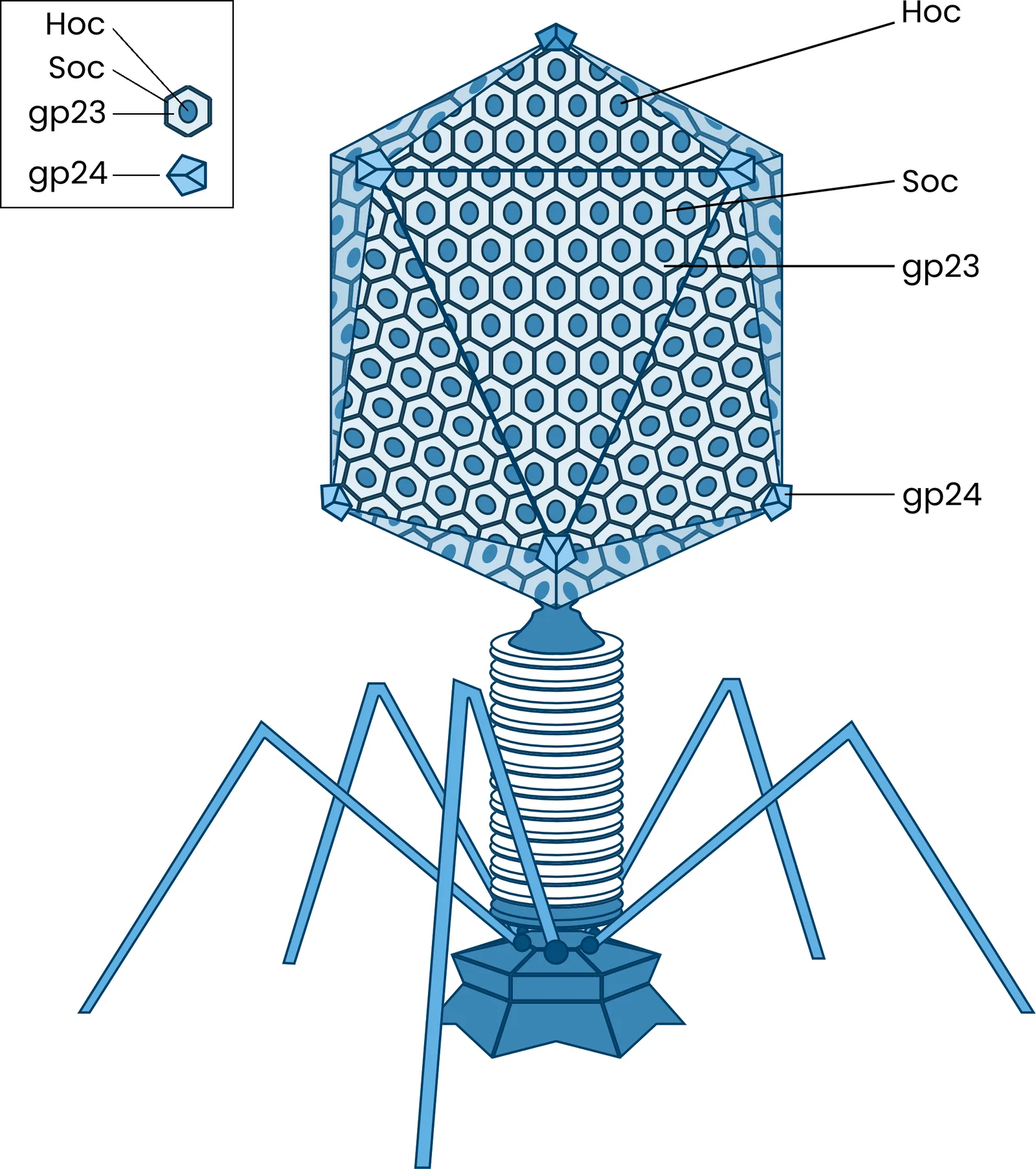
Schematic representation of the bacteriophage T4 virion. Four protein components of the head—gp23, gp24, Hoc, and Soc—and their positions within the head structure are indicated.

T4 phage is a complex virus; its virion is formed by more than 40 types of proteins (2). The virion head is a prolate icosahedron, 120 nm long and 86 nm wide, made up of four structural proteins: gp23, gp24, Hoc, and Soc (3) (Fig. 1). Gp23 (major capsid protein) forms hexamers that constitute the surface lattice, while gp24 (head vertex protein) forms pentamers on eleven non-portal vertices of the head. Gp23 and gp24 are essential for capsid assembly and maturation, and their deletions in wild-type T4 are lethal for the phage. However, lethality caused by gp24 deletions can be bypassed by certain mutations in gene *23*, which allow its protein product gp23 to form both hexamers and pentamers and to replace gp24 at the vertices of the head (4, 5). Interestingly, gp23 and gp24 share a significant homology (6) and gp24 is thought to be a relatively recent evolutionary addition to the phage virion, given the fact that it can be easily bypassed (7).

Soc and Hoc, in turn, are nonessential and they decorate the outer surface of the capsid (8). Hoc protein is located in the center of each gp23 hexamer, while Soc trimers are distributed between gp23 hexamers, serving as a molecular clamp (9, 10). It is believed that nonessential proteins are involved in improving phage fitness. Indeed, Soc is involved in stabilizing the capsid against the pressure of highly condensed genomic DNA and also in unfavorable environments (11). Experiments with the T4 mutants T4ΔSoc, T4ΔHoc and the double mutant T4ΔHocΔSoc performed in alkaline pH demonstrated that Soc stabilized the capsid in those conditions. Starting from pH 10, Soc-deficient mutants (but not Hoc-deficient mutant) were more sensitive to high pH, in comparison to wild-type T4 (11). Potential effects of Hoc on capsid stability seemed marginal (if any) and its role was postulated to involve moderating phage interactions with the mammalian immune system or mucosal surfaces (12, 13) rather than resisting physical factors.

Bacteriophage T4 infects *E. coli* cells, attaching to their surface with the long tail fibers (LTFs) and using the outer membrane protein C (OmpC) and lipopolysaccharide (LPS) as coreceptors (14). Interestingly, recent studies (15) demonstrated that the dynamics of phage-bacteria interactions in spatially structured environments—more typical in natural settings—differ significantly from those observed in well-mixed laboratory cultures. In the presence of spatial refuges, a lower level of genetic resistance to phages is observed and phenotypic resistance strategies are more relevant for bacterial survival, with heterogenous expression of the phage receptor (in the case of bacteriophage T4, namely OmpC) being one of the mechanisms. In recent years, novel approaches such as transposon insertion sequencing (16) and genome-wide loss-of-function and gain-of-function technologies (17) have been proposed for high-throughput identification of phage receptors and host factors important during phage infection. Both these studies confirmed known receptors of T4 phage and pointed to various factors potentially involved in the complex phage-host interactions. In a mature T4 virion, LTFs can adapt two conformations: retracted or extended, and individual fibers remain in a dynamic equilibrium between these two states (18). Importantly, in the retracted state LTFs are folded against the tail sheath, interacting with both the Wac protein and the capsid. Given the symmetry mismatch between the baseplate—to which the LTFs are anchored—and the icosahedral capsid, individual LTFs interact with the capsid differently, with one of the fibers interacting with the capsid edge. Therefore, it cannot be excluded that mutations and changes in the protein composition of T4 phage capsid could also affect the conformation of LTFs and, indirectly, the process of phage attachment to the host.

Specific physical factors are linked to the local microenvironment along the alimentary tract that provides optimal conditions for digestion, especially with regard to the activity of digestive enzymes that require a very specific pH range. Thus, T4-like phages within mammalian or avian microbiota are under the pressure of acidic (stomach) or alkaline (intestine) pH. Also, the body temperature of warm-blooded animals is typically higher than that of the environment. Further, phages in the gut are exposed to digestive enzymes, such as trypsin, chymotrypsins, or pepsin. This raises questions about whether the complex structure of phage capsids contributes in any way to their survival inside the GI tract of animals and humans, and—if so—what is the role of individual proteins. Interestingly, recent studies of phage interactions with human and animal systems revealed that the ways in which phages affect higher organisms go far beyond regulation of the bacterial part of microbiomes (19 – 21). However, the reverse perspective, in which phages and—to some extent—possibly also phage evolution may be affected by non-bacterial (physico-chemical) factors, including the specific natural niche of human and animal bodies (particularly the GI tract), remains underexplored.

The simplest and probably the most primitive version of the T4 phage capsid, made of gp23 only (bypass variant), is viable but not common. If so, other than gp23, potentially “dispensable” proteins may provide an evolutionary advantage to the phage in the external environment, but also when circulating inside animal or human bodies as components of their natural microbiota. In this study, we constructed and compared the physical stability of all types of T4 mutants deficient in gp24 (bypass), Soc, and Hoc, including double and triple deletions. We investigated high and low pH, elevated temperature, and digestive enzymes as physical and chemical factors that affect the T4 phage when populating gut microbiota, seeking to understand the distinct roles of individual head proteins in its survival, with the main focus on the specific conditions inside human and animal bodies. Taking into account that T4-like phages have been considered for phage therapy (22 – 24) and T4 itself could be a promising nanovector (25, 26), such data would not only expand our understanding of phage biology but also impact the field of phage application as biotechnological and medical tools, where susceptibility of phage particles and phage-based vectors to certain conditions could be either a disadvantage or a desired characteristic.

## RESULTS

### T4 phage mutants deficient in head proteins

Site-directed mutagenesis was performed on T4 phage and its previously described mutant T4∆Hoc (27) to introduce nonsense mutations into genes *24* and *soc* and, as a result, the following panel of T4 phage variants was obtained: T4∆Soc, T4∆24byp24_1, T4∆24byp24_2, T4∆Hoc∆Soc, T4∆Hoc∆24byp24, T4∆Soc∆24byp24, and T4∆Hoc∆Soc∆24byp24. ∆Soc variants were first selected by PCR (Fig. S1) and the successful introduction of nonsense mutations in the isolates was confirmed with direct sequencing. Since gp24 is essential for T4 phage capsid assembly and maturation, stable ∆24 mutations require the co-occurrence of spontaneous bypass-24 mutations in gene *23*, which compensate for the lack of gp24 (4, 5). Hence, each of the gp24-deficient mutants also harbors such bypass-24 mutations. All mutations identified in gene *23* are summarized in Table 1. Of the four identified amino acid substitutions, N381S (5) and a novel mutation, N384S, are the most relevant, as they confer the bypass-24 phenotype. The role of the identified mutations is discussed more extensively in the *Discussion* section. Briefly, bypass mutations in gp23 allow the phage to survive without gp24, which in a wild-type phage is an essential protein. Nonsense mutations in Hoc- and Soc-coding genes, in turn, simply result in the lack of these proteins in the capsid, since these two are nonessential (“decorative”) proteins. The presence or absence of gp23, gp24, Hoc, and Soc in the capsids of wild-type T4 phage and its mutants was verified using ELISA with protein-specific reference plasma samples, which confirmed that nonsense mutations in genes *24* and/or *soc* resulted in the lack of gp24 and/or Soc and therefore in a deficient composition of the head (Fig. S3).

**TABLE 1:** T4 phage mutants investigated in this study: protein composition of capsids and mutations identified in gene *23* in gp24-deficient mutants^*a*^

	gp23	gp24	Hoc	Soc	Mutations altering gp23^*b*^
T4	+	+	+	+	−
T4ΔHoc	+	+	−	+	−
T4ΔSoc	+	+	+	−	−
T4Δ24byp24_1	+ *	−	+	+	A295V (C884T) **N381S (A1142G)**
T4Δ24byp24_2	+ *	−	+	+	A295V (C884T) N384S (A1151G)
T4ΔHocΔSoc	+	+	−	−	−
T4ΔHocΔ24byp24	+ *	−	−	+	V99I (G295A) **N381S (A1142G)**
T4ΔSocΔ24byp24	+ *	−	+	−	A295V (C884T) **N381S (A1142G)**
T4ΔHocΔSocΔ24byp24	+ *	−	−	−	V99I (G295A) **N381S (A1142G)**

^*a*^ “+” or “-” indicate presence or absence of the protein, respectively; asterisks (*) indicate bypass variant of the major capsid protein (gp23).

^*b*^ Boldfacing indicates mutations in gp23 with bypass effect previously confirmed by Johnson et al. (5).

### Head vertex protein (gp24) and small outer capsid protein (Soc) improve phage resistance to body temperature of warm-blooded animals

**Fig 2:**
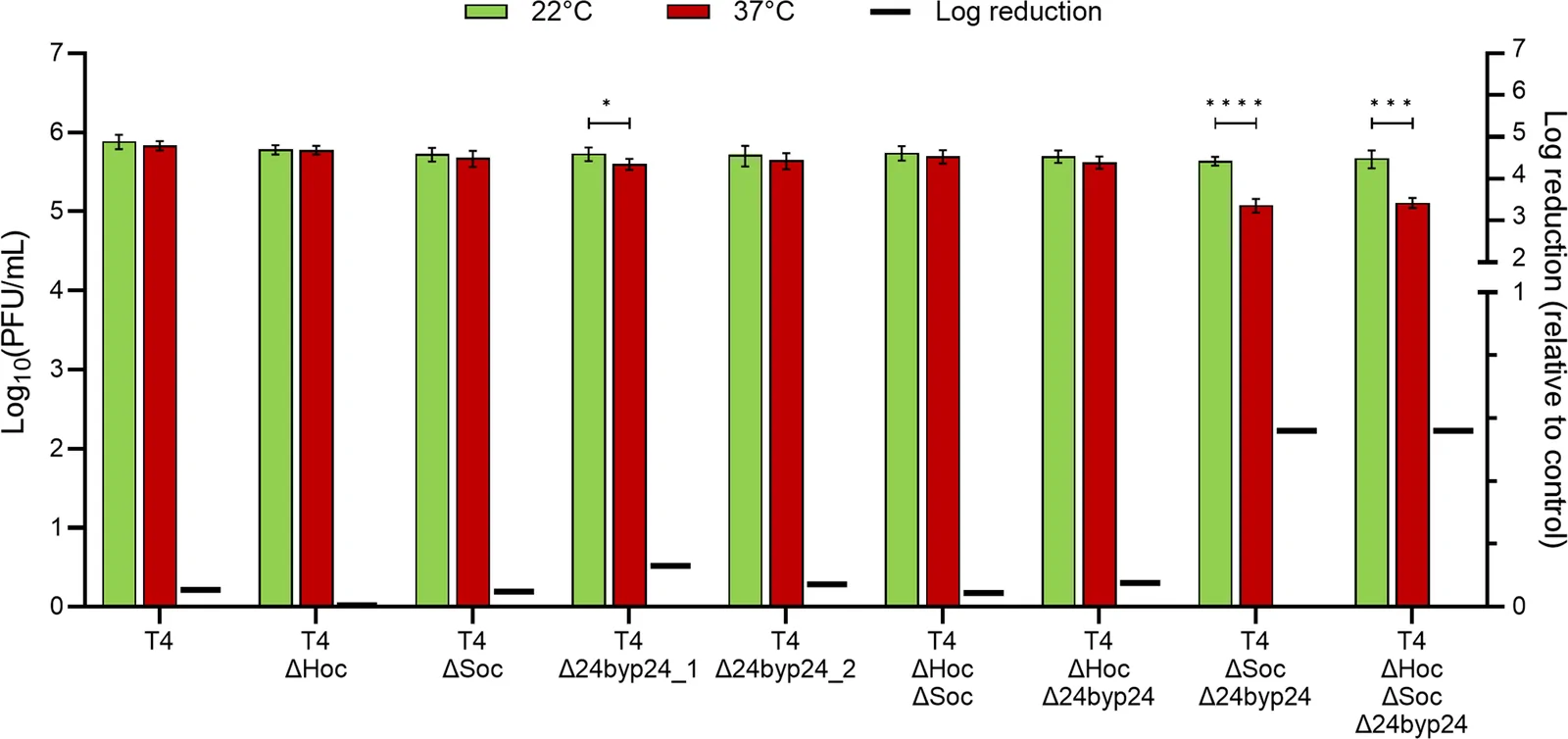
Stability of T4 phage and its mutants deficient in head proteins at 37°C. Purified phage preparations (*N* = 6) were diluted in PS to 10^6^ pfu/mL and incubated at 22°C or 37°C for 2 h. Phage titers were determined using spot plating. Log reductions were calculated relative to the control group (22°C). The experiment was repeated twice with concordant results. One representative experiment is presented. *P < 0.0243; ***P < 0.008; ****P < 0.0001 (Welch’s *t* test).

T4 phage and its head protein-deficient mutants were investigated for their susceptibility to 37°C as the approximated body temperature in warm-blooded animals. Incubation of the investigated phages at 37°C for 2 h resulted in a statistically significant decrease in phage titers in three mutants – T4Δ24byp24_1 (26% titer drop), T4ΔSocΔ24byp24 (72% titer drop), and T4ΔHocΔSocΔ24byp24 (72% titer drop)(Fig. 2). However, when exposure to 37°C was prolonged to up to 24 h (data not shown), the difference between the wild-type T4 phage and T4Δ24byp24_1 was no longer detectable, while the negative effect of temperature on the double and triple mutants deficient in head vertex (gp24) protein and Soc was still apparent. This strongly suggests that the specialized vertex protein gp24 and Soc are both involved in protecting the capsid from heat-related damage, possibly through different mechanisms. Importantly, while lack of only one of these proteins appears to have a smaller effect or no effect at all on phage survival at body temperature, simultaneously lacking both is highly detrimental.

### Head vertex protein (gp24) demonstrates remarkable thermostability

**Fig 3:**
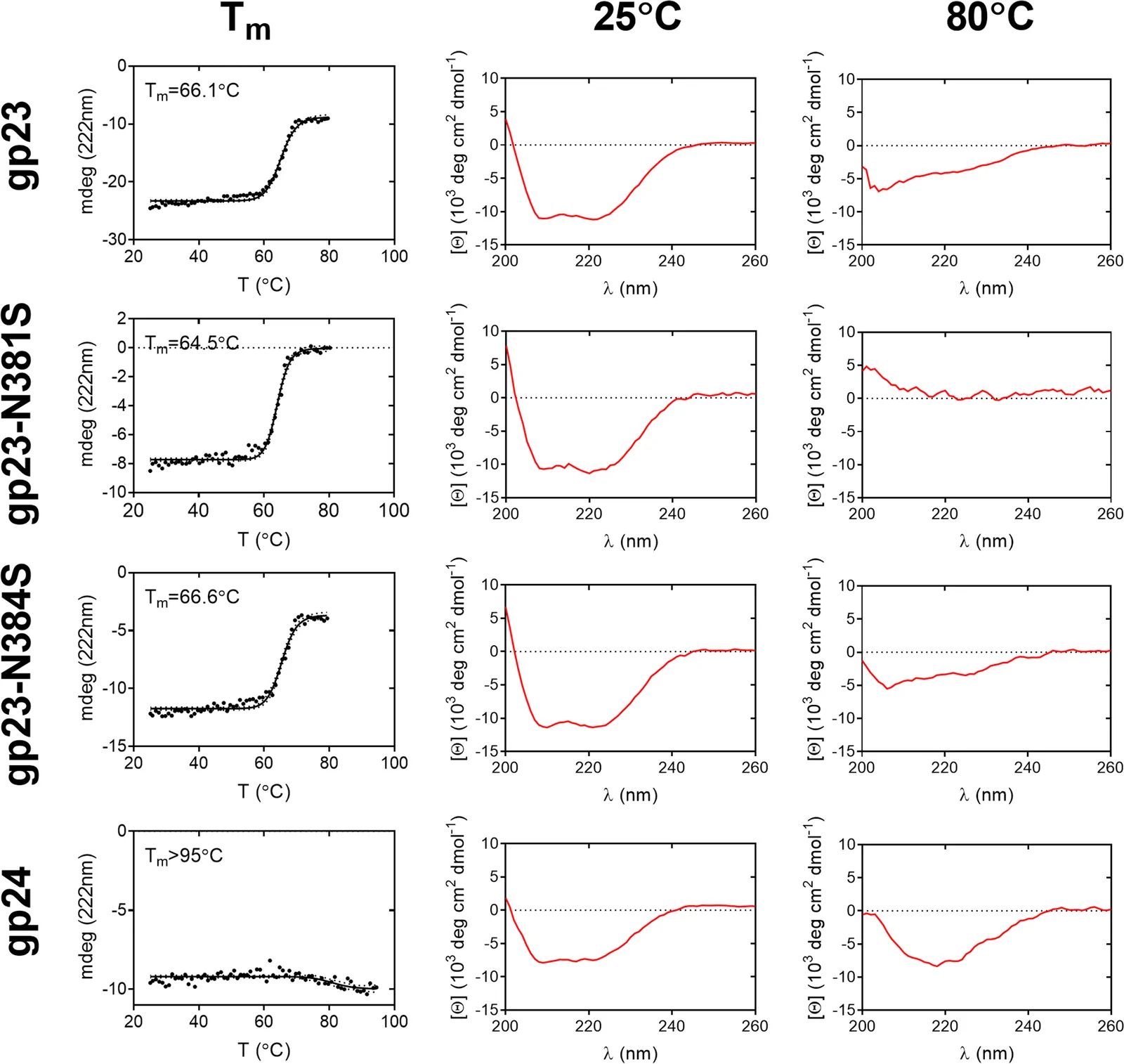
Thermal denaturation curves and circular dichroism (CD) spectra of recombinant T4 head proteins: major capsid protein gp23 and its two mutants with a single amino acid substitution N381S or N384S, and head vertex protein gp24. Protein solutions in PBS were heated in the range of 25°C–80°C (gp23 and its mutants) or 25°C–95°C (gp24); wavelength: 222 nm. CD spectra were acquired in the range of 200–260 nm at 25°C and 80°C.

Some bypass mutants of T4 phage were identified as sensitive to increased temperatures. These mutants lack protein gp24 in their capsids, but at the same time they bear characteristic bypass mutations in protein gp23, necessary for protein gp23 to form head vertices replacing gp24 (otherwise the lack of gp24 is lethal for the phage). The heads of these mutants are formed with mutated gp23, which builds both surfaces and vertices of icosahedrons, so better thermostability of the complete T4 phage (compared to its “primitive” version without gp24) can be mediated by either gp24 or the non-bypass version of gp23. To identify the molecular basis, thermostability of gp24 and gp23 was assessed by circular dichroism (CD) (folding/unfolding was assessed at 222 nm with increasing temperature). All gp23 variants showed similar denaturation curves and similar *T*_m_ values, with no apparent differences between the wild-type gp23 and its bypass mutants (Fig. 3; Table S1). In contrast, the denaturation curve of gp24 revealed unexpected, outstanding resistance of this protein to high temperatures (Fig. 3); this resistance was so strong that *T*_m_ could not be determined (Table S1). These results strongly support the conclusion that the head vertex protein gp24 likely plays a major role in maintaining the thermostability of the T4 phage capsid.

### Head vertex protein (gp24) and small outer capsid protein (Soc) are crucial for the stability of T4 phage virions in response to pH

**Fig 4:**
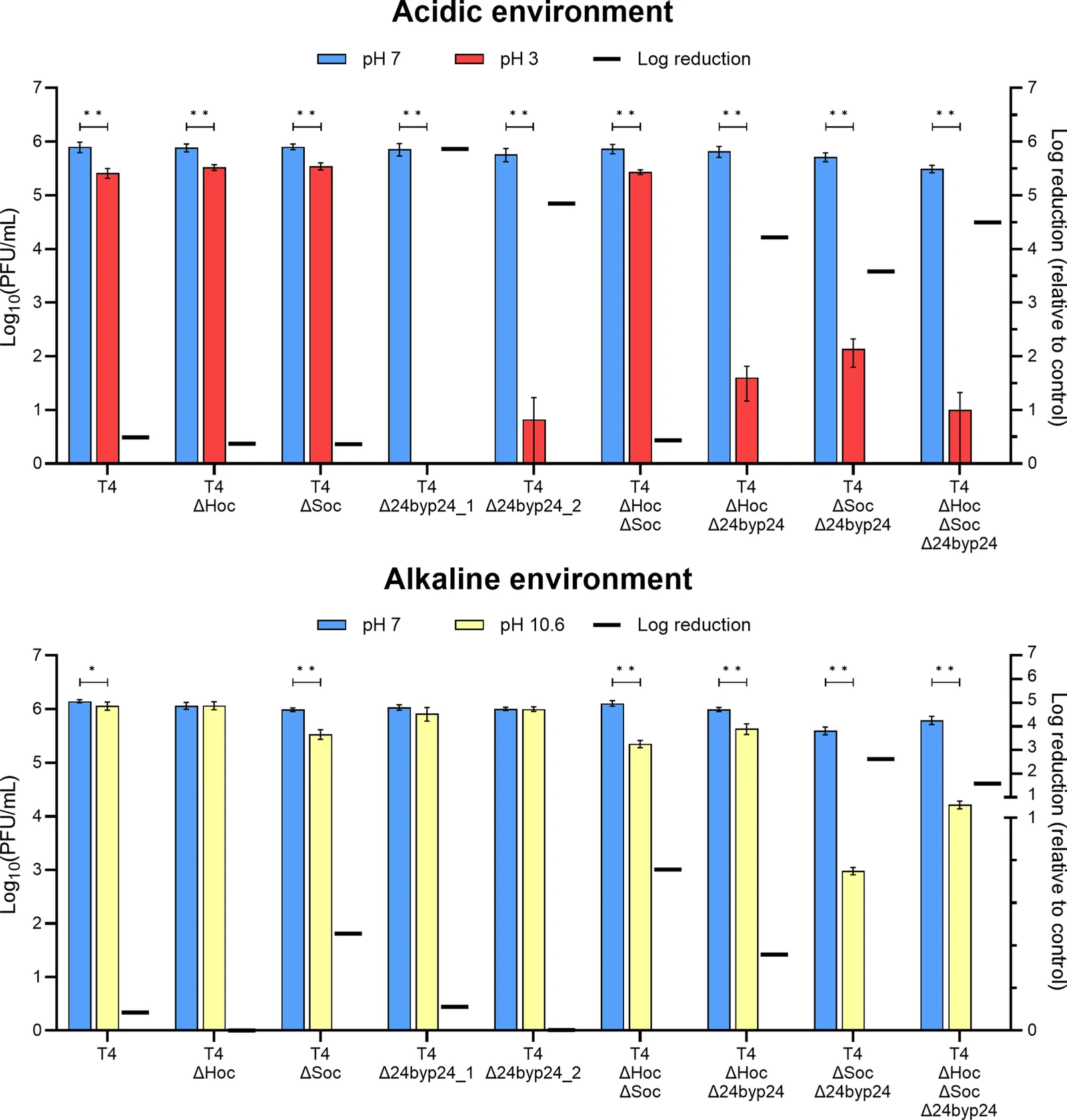
Effect of acidic, pH 3.0, and alkaline, pH 10.6, environment on the viability of T4 phage and its mutants defective in head proteins. Purified phage preparations (*N* = 6) were diluted to 10^6^ pfu/mL in PS, pH 7 and PS, pH 3 or 10.6 and incubated at 37°C for 30 min (pH 3.0) or 1 h (pH 10.6). Phage titers were determined using spot plating. Log reductions were calculated relative to the control group (pH 7). Each experiment was repeated at least twice with concordant results. One representative experiment is presented. *P < 0.05; **P < 0.005 (Mann-Whitney test).

We investigated how modifications of phage head protein composition affected T4 phage activity at acidic and alkaline pH. All variants were less stable at pH 3, but the ∆24byp24 mutants were significantly more sensitive than T4 or T4 deficient in Hoc and/or Soc, as shown in Fig. 4. The titers of ∆24byp24 mutants after 30 min incubation were three to five orders of magnitude lower than those of T4 and other gp24-positive phages, the latter ones being only moderately affected (Fig. 4, top panel). In alkaline pH, however, it was the lack of Soc that negatively affected phage viability. The sensitivity of Soc-deficient mutants to alkaline pH was further increased when the phages were also lacking gp24 (as bypass mutants) (Fig. 4, bottom panel), again demonstrating that gp24 and Soc together are crucial for the stability of T4 phage virions. Lack of gp24 or Hoc alone did not have a negative effect on phage viability at pH 10.6, yet some decrease in phage titer was observed for T4∆Hoc∆24byp24.

### Sensitivity to proteolytic digestive enzymes and bile

Phage capsids are composed of proteins and therefore on their journey along the GI tract they could potentially be prone to proteolytic activity of digestive enzymes, e.g., pepsin, trypsin, and chymotrypsins. We investigated whether the protein composition of the T4 head affected phage susceptibility to those enzymes. Wild-type T4 and its mutants were incubated with pepsin (1 mg/mL, pH 4), trypsin (2 mg/mL, pH 8, 20 mM Ca^2+^ as a cofactor), or α-chymotrypsin (3 mg/mL, pH 8, 20 mM Ca^2+^ as a cofactor), at 37°C (the optimum for these enzymes’ activity).

**Fig 5:**
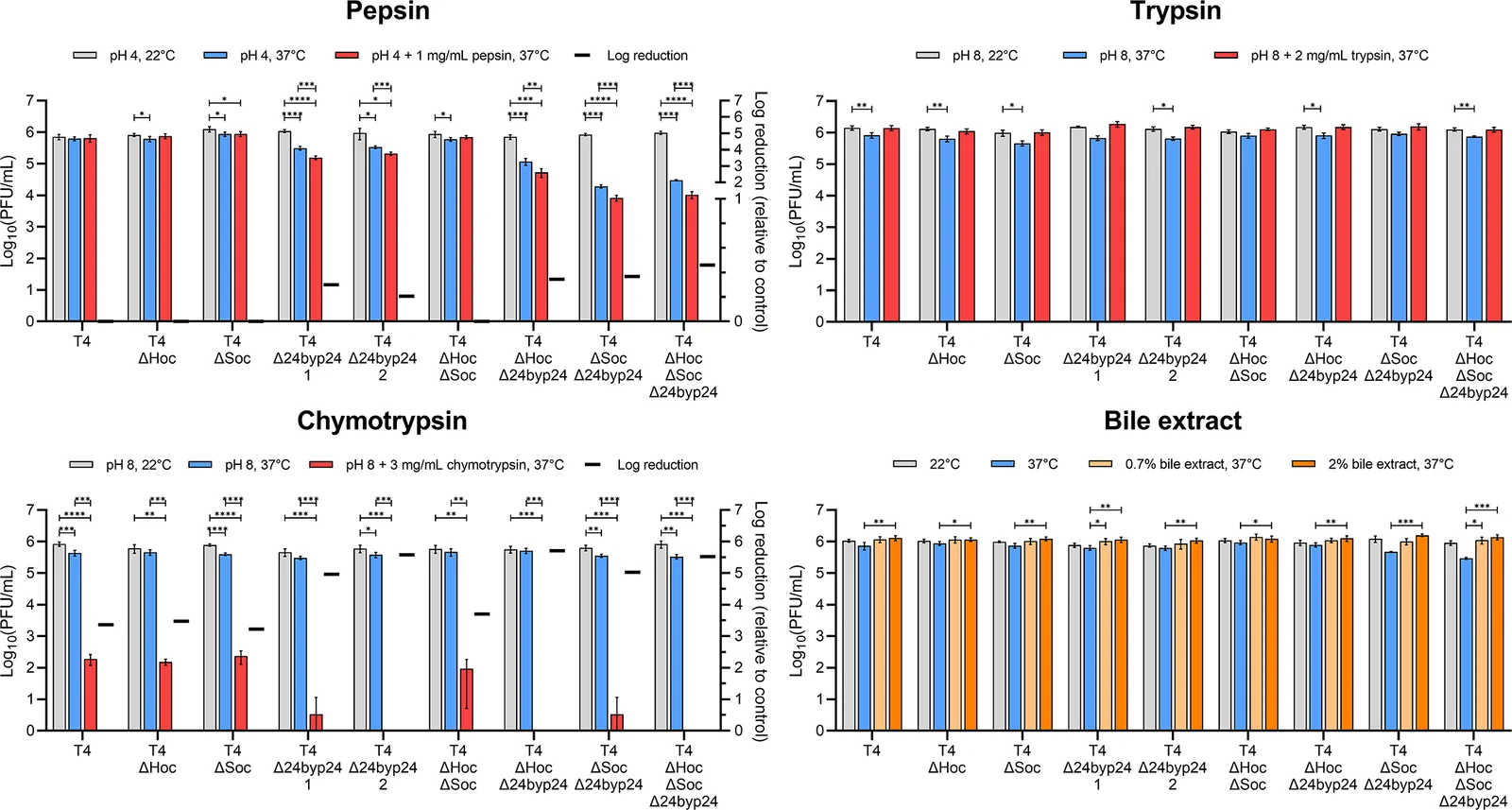
Effects of the proteolytic digestive enzymes pepsin, trypsin, and α-chymotrypsin, and bile extract on the viability of T4 phage and its mutants defective in head proteins. Purified phage preparations (*N* = 6) (10^6^ pfu/mL) were incubated in PS pH 4 or PS pH 4 with pepsin (1 mg/mL) at 37°C or in PS pH 4 at 22°C for 2 h; in PS + 20 mM CaCl_2_ pH 8 or PS + 20 mM CaCl_2_ pH 8 with trypsin (2 mg/mL) at 37°C or in PS + 20 mM CaCl_2_ pH 8 at 22°C for 2 h; in PS + 20 mM CaCl_2_ pH 8 or PS + 20 mM CaCl_2_ pH 8 with of α-chymotrypsin (3 mg/mL) at 37°C or in PS + 20 mM CaCl_2_ pH 8 at 22°C for 6 h; in PS or 0.7% and 2% porcine bile extract in PS at 37°C or in PS at 22°C for 2 h. Phage titers were determined using spot plating. Log reductions were calculated relative to the relevant control group incubated at 37°C in the absence of the investigated factor (represented with blue bars). Each experiment was repeated at least twice with concordant results. One representative experiment is presented. *P ≤ 0.0332; **P ≤ 0.0021; ***P ≤ 0.0002; ****P < 0.0001 (Brown-Forsythe and Welch ANOVA, Dunnett’s T3 multiple comparisons test).

In the case of pepsin, an acidic environment must be provided to maintain enzyme activity. Thus, optimal conditions for pepsin treatment include those previously identified as affecting ∆24byp24 mutants. To moderate the effect of acidic pH, but at the same time to provide sufficient conditions for the enzyme, the experiment was carried out at pH 4 (the upper pH limit for optimal activity of pepsin) at 37°C, with two non-pepsin-treated controls incubated at pH 4 and either 37°C or 22°C. As expected, 37°C and acidic pH affected ∆24byp24 mutants (65% to 98% titer decrease in comparison to the control incubated at pH 4 at 22°C). Nevertheless, a larger decrease in the phage titer for all gp24-deficient mutants resulted from pepsin treatment (78% to 99% titer decrease in comparison to the control incubated at pH 4 at 22°C and 38% to 65% in comparison to the non-pepsin-treated group incubated at 37°C). Again, bypass mutants deficient also in Soc protein were the most efficiently inactivated by pepsin digestion (≥99% titer decrease in comparison to the control incubated at pH 4 at 22°C and 57% to 65% in comparison to the non-pepsin-treated group incubated at 37°C) (Fig. 5; Table S2). Pepsin had no negative effect on mutants lacking Hoc or Soc, nor on the T4∆Hoc∆Soc double mutant.

Neither T4 nor any of the investigated T4 mutants appeared to be prone to proteolytic activity of trypsin, but all phages, including wild-type T4, were found to be sensitive to α-chymotrypsin (Fig. 5), with gp24-deficient phages again being significantly more affected.

No decrease in phage titers was observed after incubation with either 0.7% or 2% bile extract for any of the mutants. Bile concentration of 0.7% corresponds to that found in human GI tract (28). While some mild effects of bile components may have been undetectable *in vitro* at a physiological concentration, using a nearly threefold higher bile content likely would have made any such effects more apparent. Therefore, this strongly suggests that bile components do not have a negative effect on bacteriophage T4 regardless of the protein composition of the capsid (Fig. 5).

## DISCUSSION

Out of the four proteins comprising the head shell of a mature T4 phage, only gp23 and gp24 are essential components. While gp23 hexamers form the surface lattice, gp24 builds 11 pentameric vertices of the bacteriophage T4 capsid. These two proteins share ~21% amino acid sequence identity (6) and 31% sequence similarity (29) and gp24 is postulated to be a gp23 derivative, originating from a gene duplication and subsequent sequence divergence and optimization (30). Moreover, specialized vertex protein likely is a relatively recent evolutionary addition, given the fact that spontaneously occurring specific mutations in gp23 quite easily allow for a functional substitution of gp24 (7), bypassing the otherwise lethal effect of gp24 deficiencies. Johnson et al. (5) identified N381S substitution in gp23 as a bypass-24 mutation sufficient to confer a bypass phenotype. Although a similar N384S substitution has not been described to date, given the consistent nature of the substitutions, where asparagine is substituted with serine, and the close proximity of this novel mutation to the originally described position, we propose to include N384S substitution identified in this study in the list of bypass-24 mutations. Considering the fact that another verified bypass-24 mutation, A387T (5), although involving an amino acid substitution of different nature, is located in close proximity, this region of gp23 appears to be important for gp23 structural function. This was in fact recently confirmed by Fang et al. (31), who demonstrated that N381S and A387T are located within a short α-helix of the A domain of gp23, at the interface of adjacent protein subunits within the same capsomer and are involved in modulating intersubunit interactions.

All gp24-deficient mutants selected in this study carry either N381S or N384S substitution. However, these mutations are accompanied by A295V or V99I substitutions (Table 1). Johnson et al. (5) described a different mutation at position 295, where alanine was replaced with threonine, and concluded that it does not confer a bypass-24 phenotype, nor does another closely located mutation, T296A. This strongly suggests that the A295V substitutions observed in the mutants investigated herein do not confer the bypass phenotype. V99I, on the other hand, was involved in conferring the bypass-24 phenotype when accompanied by A126T mutation, yet neither of these two substitutions was sufficient when introduced alone. In our study, V99I mutation was accompanied by N381S, which is a sufficient bypass-24 mutation itself; therefore, the exact role of V99I is not clear, but most likely it has no important role in the definition of the phenotype here.

We have found bypass-24 mutants of T4 simultaneously deficient in Soc protein to be significantly more sensitive to increased temperature than wild-type phage or other types of mutants. Surprisingly, we observed sensitivity to relatively mild conditions: these phages were negatively affected by a temperature of 37°C. Importantly, all variants were stable at 22°C. T4 phage life cycles are dependent on *E. coli* bacteria, which are common symbionts of mammals or birds. Since T4-like phages belong to mammalian or avian GI tract microbiota, they are constantly exposed to temperatures exceeding ambient levels. The observation that the “primitive” version of the phage capsid is sensitive to a temperature of 37°C highlights the role of gp24 and Soc in phage survival when inside animal and human bodies. Importantly, selection for this adaptation may of course have come from many other sources, including temperature and pH variations that may occur in the external environment. Of note, while lack of either gp24 or Soc alone does not result in a substantial sensitivity to mildly higher temperatures, simultaneously lacking both proteins is highly detrimental. This points to the evolutionary advantage of the acquisition of a specialized vertex protein and supports the role of Soc as a molecular glue stabilizing the phage capsid in response to thermal denaturation (32).

Since the lack of gp24 itself is lethal for the phage and it needs to be compensated with bypass mutations in gp23 to keep the phage viable, differences in phage sensitivity to temperature and other factors may result directly from the absence/presence of gp24, but alternatively they may also result from the altered amino acid sequence of gp23. The bypass (“primitive”) version of gp23 might have overall lower stability, thereby leading to impaired resistance to environmental stress of the whole phage head. Therefore, we compared the thermostability of isolated recombinant proteins gp24 and gp23, and two bypass mutants of gp23 (Fig. 3). We observed that bypass mutations N381S or N384S in gp23 did not affect its thermostability. At the same time we observed that gp24 exhibited spectacular resistance to high temperature: denaturation was not observed even at 95°C and *T*_m_ could not be determined (Fig. 3; Table S1). Thus, we conclude that differences in thermosensitivity between T4 phage and its gp24-deficient mutants can be attributed to the absence/presence of gp24 rather than amino acid substitutions in gp23, rendering the gp24-deficient phages more prone to environmental stress, including the conditions inside the bodies of warm-blooded animals where phages exist within normal intestinal microbiota.

In addition, we noted that the detrimental effect of temperature was counteracted by the introduction of Ca^2+^ ions to the phage’s microenvironment. This protective effect of Ca^2+^ was, however, also applicable to wild-type T4 (Fig. S4); thus, a specific effect of Ca^2+^ on the bypass mutants was not observed, and the effect is rather general for T4-like phages. Also, luminal concentrations of Ca^2+^ ions in the GI tract are strongly dependent on dietary calcium intake and potentially highly variable. Therefore, the potential protective role of Ca^2+^ in the GI tracts of animals and humans is not certain, especially considering the fact that many phages rely on the presence of divalent cations for stability and propagation. In nature, calcium is considered an easily available element as it is among the most abundant cations in water and soil. Nevertheless, concentrations of Ca^2+^ ions vary depending on the environment, typically reaching ~10 mM in open ocean water (33) and ranging between 0.01 and ~1.9 mM in freshwater, with the average at ~0.1–0.35 mM and much higher concentrations observed in the vicinity of carbonate rocks (34). Calcium concentrations in the soil solutions were reported to reach up to 20 mM (35) and strongly depend on the type of soil.

The environment of the GI tract is also characterized by fluctuations in pH, typically from strongly acidic contents of the stomach to more alkaline conditions in further sections (detailed conditions differ between species), and by the presence of digestive enzymes. Proteolytic enzymes such as pepsin, trypsin, and chymotrypsins may potentially affect the phage, considering the proteinaceous nature of phage capsids. All these factors constitute a specific and diverse environmental niche that may negatively affect phage particles. Although ∆Soc mutants of bacteriophage T4 have previously been reported as more susceptible to extreme pH conditions (11), those stability tests have only been performed at alkaline pH, and more importantly they did not include gp24 bypass mutants. In this study, we confirmed that a lack of Soc negatively affected phage viability in alkaline pH, and observed that sensitivity to an alkaline environment was further increased when Soc-deficient mutants were also lacking gp24 (Fig. 4). On the other hand, exposure of phages to acidic pH caused massive neutralization of ∆24byp24 mutants regardless of the presence or absence of Hoc and/or Soc. These results show that the molecular bases for T4 phage resistance to acidic and alkaline pH are likely fundamentally different. The role of Soc protein as a molecular glue stabilizing the capsid in extreme environmental conditions may in fact be manifested in alkaline conditions, while in an acidic environment it is gp24 rather than Soc that is vitally important for T4 phage viability. Of note, we have previously found that the chemical stability of gp24 was also markedly higher than that of gp23 (36). Thus, the molecular switch from gp23-only capsids to capsids built with both gp23 and gp24 could have been promoted by both increased temperatures and acidic conditions—primarily in the external environment or perhaps also in the GI tracts.

Bacteriophages, even those relatively closely related, differ in their susceptibility to proteolytic degradation by digestive enzymes. To list a few examples, studies on other *E. coli* myoviruses—T2 (a close relative of T4) and P1 (37)—and an *E. coli* podovirus CA933P (38) revealed that T2 phage was inactivated by high concentrations of chymotrypsins, but not trypsin, while P1 was rapidly inactivated by trypsin and more slowly by chymotrypsins, with Δ-chymotrypsin-mediated inactivation being more potent than that of other three chymotrypsins. Free, non-encapsulated CA933P was relatively quickly inactivated by pepsin, but was not affected by bovine intestinal proteases—trypsin and α-chymotrypsin—despite a long incubation period of 24 h. However, it is unclear whether microenvironmental conditions were optimal for the proteolytic activity of intestinal proteases (normal saline solution, pH 7.2, no indication of the presence and concentration of calcium ions). Additionally, as many phages are highly sensitive to acidic pH, evaluation of pepsin-mediated inactivation is often challenging. Even though the data regarding phages and digestive enzymes are somewhat limited, inactivation by proteolytic enzymes is likely phage-specific and, therefore, in terms of phage resistance to proteolysis there is no universal pattern that could be applied to bacteriophages, even those of a similar morphotype or specificity. When it comes to T4 phage in particular, Ishii and Yanagida (11) tested T4ΔSoc, T4ΔHoc, or T4ΔSocΔHoc mutants and reported no differences in their susceptibility to digestion by trypsin in comparison to wild-type T4. We observed herein that while neither T4 nor any of its mutants were inactivated by trypsin, all of them were sensitive to the proteolytic activity of α-chymotrypsin, with a more severe titer decrease again being observed for gp24-deficient phages (Fig. 5). Also, in the case of pepsin, lack of gp24 rendered the phages prone to proteolytic digestion, while those with intact gp24 remained unaffected. Bypass mutants deficient also in Soc protein were inactivated more efficiently, which again indicates that both gp24 and Soc are involved in improving and maintaining the stability of the T4 phage capsid.

The key to understanding the observed differences in susceptibility to proteolytic digestion likely lies in the spatial architecture of phage capsids and/or the substrate specificity of these enzymes. Trypsin, α-chymotrypsin, and pepsin all display distinct substrate specificity—trypsin is highly specific and favors basic residues like lysin and arginine, while α-chymotrypsin selectively catalyzes the hydrolysis of peptide bonds after aromatic amino acids (tyrosine, phenylalanine, and tryptophan) and leucine, with secondary hydrolysis also occurring after methionine, isoleucine, serine, threonine, valine, histidine, glycine, and alanine (39, 40). Pepsin is reported to be much less specific than trypsin and other proteases (40), but it was reported to favor bulky hydrophobic amino acid residues—in general, it preferentially cleaves after phenylalanine and leucine, yet for many other amino acids the cleavage probability depends greatly on the adjacent residues (41). When interactions between individual protein elements building the phage capsid are added to the equation, some amino acids may remain “hidden,” while at the same time other residues are exposed and available for cleavage by proteolytic enzymes. It is clear that bypass-24 mutations in gp23 greatly affect how monomers of this protein interact with one another to form oligomers—hexamers and also pentamers, not observed in the case of regular gp23. Therefore, it is likely that some amino acid residues that would have been protected from the proteolytic enzymes in regular gp23 hexamers are rendered more exposed to the environment in these pentameric structures and perhaps also in the mutated hexamers, as slight shifts in conformation could occur compared to wild-type gp23. This could be even more relevant in ∆Soc∆24byp24 variants, when the molecular net of Soc trimers normally located between the gp23 hexamers is also absent. However, these speculations would require experimental confirmation. On the other hand, introduction of additional serine residue in place of asparagine in the investigated bypass mutants (N381S or N384S) could theoretically provide an additional cleavage site for the digestive enzymes. However, this explanation seems less plausible as this region of gp23 is generally not well exposed (31).

In addition to mammalian physiology-related conditions and their impact on T4 phage variants, we observed that some of the investigated variants were sensitive to technological processing, specifically purification and concentration with the hollow fiber system. This system includes the use of forced flow and pressure, thus potentially affecting phage particles as a physical factor. Surprisingly, the most sensitive and unstable were mutants bearing two deletions at the same time: Δ24byp24 and ΔHoc (Table S3). This further supports the key role of gp24 in resisting physical conditions by the phage; here, however, it is supported by the Hoc protein. We hypothesize that this may result from stabilizing effects that Hoc potentially exerts on gp23 hexamers, particularly under external pressure. These observations may shed new light on the possible role of Hoc—previously considered only marginally involved in providing stability—again highlighting how different elements in the complex structure of T4 capsid contribute to its overall fitness: while Soc trimers form a molecular cage that may reinforce the phage head against the internal pressure of tightly packed genetic material (10, 30), resisting external pressure appears to require a different mechanism. External pressure and flow conditions may to some extent resemble gut conditions in the area of intensive peristaltic movements, but this problem requires further studies. We propose this observation as evidence of how capsid proteins directly determine phage stability under common laboratory procedures like purification and concentration, which may have a practical impact on the development of phage processing methods as well as in the context of application of modified phage capsids—and particularly T4 capsid—as biotechnological (42) and medical tools, e.g., as vaccine platforms and artificial viral vectors (25, 26, 43). Hoc protein, however, has already been proposed as potentially mediating phage interactions with the mammalian immune system (12) and with mucosal surfaces (44), which strongly suggests Hoc contribution to improved phage survival inside the gut.

**Fig 6:**
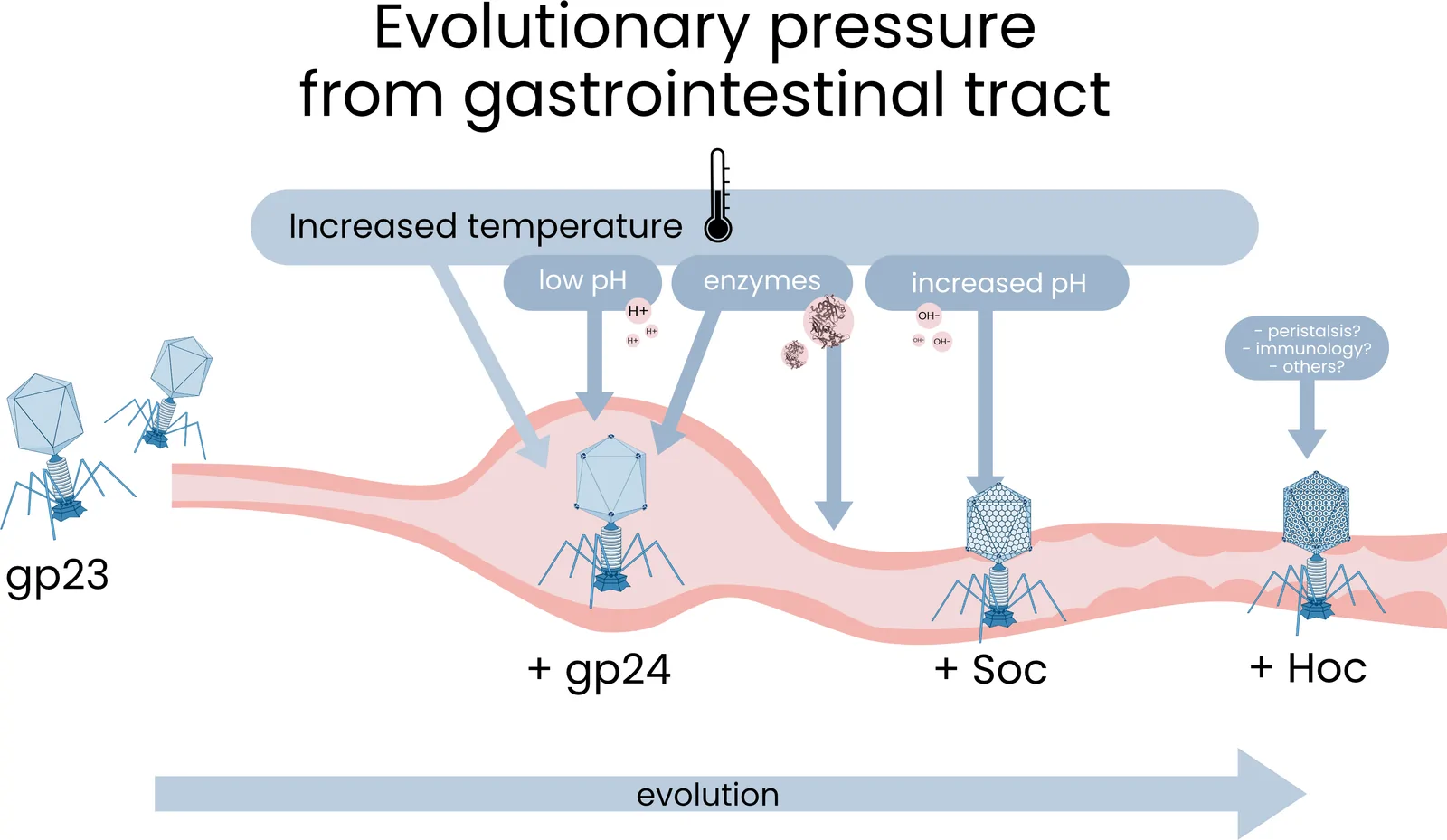
Schematic representation of the proposed gastrointestinal tract-related factors that may have contributed to the selection pressure on T4 phage.

Factors such as temperature or low/high pH are not exclusively specific to the conditions inside animal bodies, but rather remain general characteristics of any given environmental location. Also bacterial hosts of T4-like phages are not restricted to the gut. Although *E. coli* was long believed to be unable to replicate in the extraintestinal environment (45), some specific naturalized strains have recently been reported to survive and potentially reproduce in soil, sediments, or in association with macrophytic algae and periphyton in aquatic ecosystems (46). However, their long-term survival is strongly affected by environmental conditions and their genotypes are distinct from those of animal-origin *E. coli*. Comeau and Krisch (29) analyzed the T4 phage superfamily regarding the prevalence of head proteins. Homologs of all four proteins were only identified in phages very closely related to T4, while the most distant group comprising phages infecting cyanobacteria and thermophilic eubacteria (typically environmental hosts) had isometric capsids and no homologs of gp24, Hoc, or Soc. Nevertheless, the evolution of T4 phage capsid could have been influenced mostly by environmental niches very distinct from those of animal bodies. Alternatively, T4 phage capsid evolution could have been—at least partially—directed or co-directed by the microenvironment of the GI tracts of warm-blooded animals, where its host *E. coli* is abundantly present and thriving. This alternative scenario is demonstrated in Fig. 6, highlighting the GI tract-related factors and their hypothetical influence.

Regardless of the exact source of selection pressure, our observations demonstrate the key role of T4 phage capsid composition in phage resistance to a variety of conditions, all of which are also highly relevant in the context of phage presence in the GI tracts of animals and humans. Since it is believed that the specialized vertex protein gp24 evolved as a result of gene *23* duplication and subsequent optimization, what we now consider as bypass mutations in gp23 could very well reflect a less specialized, initial variant of gp23. In this scenario, a phage lacking the specialized vertex protein would in fact represent a more ancestral and “primitive” variant. We demonstrated that such simple and “primitive” structure of the phage capsid, without gp24 that was acquired relatively late in evolution, is significantly less stable at low pH and more sensitive to inactivation by digestive enzymes. Moreover, the nonessential protein Soc—the least prevalent capsid structural protein among the T4 phage superfamily (29)—and gp24 both play major role in T4 phage stability at warmer temperatures, again pointing to the improved fitness of the T4 phage compared to its ancestral variant, most likely lacking nonessential head proteins. Thus, we postulate that the key role of gp24 in phage structure is to provide the capsid with better resistance to harsh environmental conditions, including those that affect T4 phage along the GI tract. Considering that T4-like phages propagate on bacterial symbionts in the gut (*E. coli*) and are common and abundant elements of the gut microbiota, we also hypothesize that the specific conditions inside animal/human bodies could partially contribute to the evolutionary pressure on T4-like phages, together with the external environment, shaping the structure of phage virion and promoting stabilization of the most optimal phenotypes.

## MATERIALS AND METHODS

### Bacteriophages

Bacteriophage T4 was purchased from the American Type Culture Collection (ATCC) (Rockville, Maryland, USA). Its mutant HAP1 (T4ΔHoc) was previously selected and described at the Hirszfeld Institute of Immunology and Experimental Therapy, Polish Academy of Sciences (HIIET PAS) (27) and is a part of the Polish Collection of Microorganisms (HIIET PAS, Poland). HAP1 mutant bears the C496T transition in the *hoc* gene (Table 2), which results in an ochre stop codon at 44% of the protein length and therefore the mature HAP1 bacteriophage lacks functional Hoc protein on its capsid. Phages T4 and T4∆Hoc served for the construction of other mutants with altered head composition, as described subsequently.

**TABLE 2:** Fragments of nucleotide sequences of wild-type (wt) T4 phage genes *24*, *hoc,* and *soc* and their mutated variants with nonsense (*) mutations, either spontaneous (∆Hoc) or introduced into phage genomes by site-directed mutagenesis (∆gp24 and ∆Soc), and corresponding changes in the amino acid sequence of the protein products

T4 strain	Nucleotide and amino acid position	Sequence *^a^*								
gp24										
wt	289 97	AAA K	GGC G	GAT D	TTA L	TTC F	AAA K	TAT Y	AAT N	AAT N
∆gp24	289 97	AAA K	**TAG** *	GAT D	TTA L	TTC F	AAA K	TA**G** *	AAT	AAT
Hoc										
wt	481 161	ATT I	AAA K	TGC C	GTA V	GCC A	CAA Q	GTA V	ACC T	GCG A
∆Hoc	481 161	ATT I	AAA K	TGC C	GTA V	GCC A	**T**AA *	GTA	ACC	GCG
Soc										
wt	1 1	ATG M	GCT A	AGT S	ACT T	CGC R	GGT G			
∆Soc	1 1	ATG M	GCT A	**TAG** *	A**TA** I	**GAG** E	**TAG** *			

^*a*^ Boldfacing indicates changes in the nucleotide sequence, the resulting STOP codons are underlined.

### Bacterial strains

All phages were propagated on an *E. coli* B host from the Polish Collection of Microorganisms (HIIET PAS, Poland). *E. coli* DH5α, genotype: F^–^ Φ80*lacZ*ΔM15 *Δ(lacZYA-argF)U169 recA1 endA1 hsdR17*(r_K_^–^, m_K_^+^) *phoA supE44 thi-1 gyrA96 relA1 λ* ^*-*^ (Invitrogen) and *E. coli* DP50 (later referred to as supF), genotype: F^−^ *fhuA53 dapD8 lacY1 or Δ(cod-lacI)6 glnX44*(AS) *Δ(gal-uvrB)47 λ^−^ tyrT58*(AS) *gyrA29*(NalR) *ΔthyA57 hsdS*, kindly supplied by Professor Andrey Letarov (Russian Academy of Sciences, Moscow) were used for *in vivo* recombination cultures and *E. coli* supF also for selection of gp24-deficient phages. The supF strain is a nonsense suppressor strain that carries a *tyrT* mutation in the anticodon region of tRNA^tyr^ (GUA → CUA), which allows for the incorporation of tyrosine at the amber termination codon, therefore preventing premature termination of the protein production at the site of nonsense mutation.

### Site-directed mutagenesis and selection of mutants from the overall phage progeny

Nonsense mutations were introduced to phage genomes using site-directed mutagenesis and *in vivo* recombination cultures.

#### gp24-deficient phages

A fragment of the T4 phage *24* gene was amplified in a PCR with the mutagenizing forward primer 5ʹ-TATCAAATACCTTAACCCAGACAACGAATTTACATTTAAAACTGGTGCTACTTACGCTGGCGAAGCTGGATATGTAGACCGAGAACAAATCACAGAATTAACAGAAGAGTCTAAATTAACTCTCAATAAATAGGATTTATTCAAATAGAATAATATCG-3ʹ and the reverse primer 5ʹ-AACCAGCCTGATGCAGCAAGAAT-3ʹ to implement two nonsense codons into the PCR product (Table 2), subsequently cloned into pGEM-T Easy vector (Promega).

Gp24-deficient mutants must contain a bypass mutation in gp23 to compensate for this deficiency (otherwise lethal); bypass mutations are known to occur spontaneously. To construct these mutants, a two-step procedure was used. First, nonsense mutations were introduced into gene *24*, and then the phage was subjected to selection to promote bypass mutations in gene *23*. Briefly, the nonsense mutation suppressor strain *E. coli* supF was transformed with a pGEM-T Easy plasmid construct with a fragment of T4 phage gene *24* with nonsense codons (Table 2). The recombination culture was then performed, where *E. coli* supF transformed with the construct was infected with the phage to allow for homologous recombination between the plasmid and the phage genome. The resulting phage lysate was filtered through a 0.22 µM syringe filter and plated on double-layer agar with *E. coli* supF as a host; from this step on, a non-transformed *E. coli* supF strain was used. Single plaques were then transferred to corresponding sections of double-layer agar plates with either *E. coli* B or *E. coli* supF. Plates were incubated overnight and analyzed to identify sections where lysis was observed on the nonsense suppressor strain *E. coli* supF, but not on *E. coli* B. Phage isolates collected from such sections were propagated on *E. coli* supF (37°C, 8 h, vigorous shaking), lysates were filtered through 0.22 µM syringe filters and nonsense mutations in gene *24* were confirmed with direct sequencing (Genomed S.A., Poland). Confirmed gp24-deficient isolates were plated on double-layer agar plates with *E. coli* B as a host to provide selection for spontaneous bypass mutations in gene *23*. Single plaques were isolated and propagated on *E. coli* B (37°C, 8 h, vigorous shaking), and the lysates were filtered through 0.22 µM syringe filters. Reverse mutations of gene *24* were ruled out by direct sequencing of gene *24*, and gene *23* was sequenced (Genomed S.A., Poland) to identify bypass mutations.

#### Soc-deficient phages

pMA plasmid construct bearing a fragment of the T4 phage *soc* gene with nonsense codons at the beginning of the gene (Table 2) was synthesized *de novo* in GeneArt (Thermo Fisher Scientific) and used for the transformation of *E. coli* DH5α chemocompetent cells. The recombination culture was then performed, where *E. coli* DH5α transformed with the construct was infected with the phage to allow for homologous recombination between the plasmid and the phage genome. The resulting phage lysate was filtered through a 0.22-µM syringe filter and plated on double-layer agar with *E. coli* B as a host. Single plaques were isolated and suspended in sterile PBS. Unlike gp24, Soc is a nonessential protein; therefore, a selection method based on a nonsense suppressor and a wild-type host strain described previously could not be applied to identify the mutants. A PCR-based approach was used instead—for each isolate two reactions were run in parallel: one with a primer complementary to wild-type *soc* (5ʹ-GAAAACCTGTATTTTCAGGGCAGCAGCAGCATGGCTAGTACTCGCGG-3ʹ) and the other to *soc* with the mutations (5ʹ-AATTACATGGCTTAGATAGAGTAG-3ʹ) and a common reverse primer (5ʹ-GGGGACCACTTTGTACAAGAAAGCTGGGTCCTAACCAGTTACTTTCCAC-3ʹ). An example result is presented in Fig. S1 (Supplemental Materials). The results were confirmed with direct sequencing (Genomed S.A., Poland). Mutants were propagated on *E. coli* B (37°C, 8 h, vigorous shaking), and the lysates were filtered through 0.22 µM syringe filters.

### Bacteriophage preparations

All phages were propagated on *E. coli* B. To prepare crude phage lysates, nutrient broth was inoculated with a 3-h bacterial host culture and phages. Flasks were incubated at 37°C with vigorous shaking for 8–10 h, and then transferred to 4°C for 2 days to clarify. Phage lysates were then centrifuged at 8,000 rpm; the supernatants were filtered through 0.22 µM Millipore membrane filters (Merck Millipore) and purified using size exclusion chromatography on Sepharose 4B (Sigma-Aldrich) as described previously (47). Phage titers in lysates and purified preparations were determined using serial dilutions and spot plating.

### Phage protein composition verification in ELISA

MaxiSorp flat-bottom 96-well plates (Nunc, Thermo Scientific) were coated with purified phage preparations, 5 × 10^9^ pfu/mL, overnight. For the detection of Soc, phage preparations were incubated at 80°C for 30 min prior to the coating to disrupt the structure of the capsid and make Soc available for interactions with Soc-specific antibodies. For all the other proteins, intact, non-temperature-treated phage preparations were used. Plates were then washed five times with PBS and blocked with fivefold diluted SuperBlock Blocking Buffer (Thermo Scientific) at room temperature (RT) for 45 min. The blocking solution was then removed, and the plates were washed five times with PBS with 0.05% Tween 20. Protein-specific mice plasma samples (anti-gp23, anti-gp24, anti-Hoc, anti-Soc, and anti-albumin as a negative control) diluted (200-fold, except for anti-Soc, which was diluted 100-fold) in PBS were then added to wells at 100 µL per well and the plates were incubated at 37°C for 2 h. Each phage/plasma combination was investigated in duplicate. Subsequently, plates were again washed five times with PBS with 0.05% Tween 20 and 100 µL of horseradish peroxidase-conjugated goat anti-mouse IgG (Jackson ImmunoResearch Laboratories) was added to wells and incubated for 1 h at RT in the dark. The antibody solution was then removed, and the plates were washed five times with PBS with 0.05% Tween 20. TMB (50 µL/well) was used as a peroxidase substrate according to the manufacturer’s instructions (R&D Systems) and incubated for 30 min. Finally, 25 µL of 2N H_2_SO_4_ was added to stop the reaction, and the absorbance was measured at 450 nm (main reading) and normalized by subtracting the background absorbance at 570 nm.

### Electron microscopy

High-titer phage suspensions for visualization of phage particles were obtained by ammonium acetate precipitation (48). Then, 50 µL of phage preparations was deposited on nickel formvar/carbon coated transmission electron microscopy (TEM) grids (400-mesh) and incubated for 1 min. After the excess liquid was removed, the samples were stained with 0.5% uranyl acetate and observed with a Zeiss EM900 transmission electron microscope. The general morphology of phage virions was examined, and no significant differences were found (Fig. S2).

### Temperature sensitivity

Purified phage preparations (10^6^ pfu/mL) were incubated in 0.9% NaCl (physiological saline, PS) at 22°C or 37°C for 2 h. To investigate the effect of Ca^2+^, purified phage preparations (10^6^ pfu/mL) were incubated in PS at 22°C (control) or 37°C or in PS supplemented with 5 mM or 20 mM CaCl_2_ at 37°C for 24 h. Samples were then serially diluted, and phage titers were determined using the spot plating technique. The molar concentration of sodium ions in the PS used as a primary microenvironment for phage stability testing is ~150 mM, which had been previously indicated by Szermer-Olearnik et al. (49) as a high-ionic strength environment, preventing clustering of phage particles into aggregates.

### Susceptibility to acidic and alkaline pH

Purified phage preparations (10^6^ pfu/mL) were incubated in physiological saline (PS) at neutral (pH 7) and either acidic (pH 3, 30 min) or alkaline pH (pH 10.6, 1 h) at 37°C. Samples were then serially diluted, and phage titers were determined using the spot plating technique.

### Susceptibility to bile

Purified phage preparations (10^6^ pfu/mL) were incubated in PS or 0.7% and 2% porcine bile extract (Sigma-Aldrich) in PS at 37°C or in PS at 22°C for 2 h. Samples were then serially diluted, and phage titers were determined using the spot plating technique.

### Susceptibility to proteolytic activity of digestive enzymes

Purified phage preparations (10^6^ pfu/mL) were incubated in PS pH 4 or PS pH 4 with 1 mg/mL of pepsin (from porcine gastric mucosa, Sigma-Aldrich) at 37°C or in PS pH 4 at 22°C for 2 h.

Purified phage preparations (10^6^ pfu/mL) were incubated in PS + 20 mM CaCl_2_ pH 8 or PS + 20 mM CaCl_2_ pH 8 supplemented with 2 mg/mL of trypsin (Sigma-Aldrich) at 37°C or in PS + 20 mM CaCl_2_ pH 8 at 22°C for 2 h.

Purified phage preparations (10^6^ pfu/mL) were incubated in PS + 20 mM CaCl_2_ pH 8 or PS + 20 mM CaCl_2_ pH 8 supplemented with 3 mg/mL of α-chymotrypsin (Sigma-Aldrich) at 37°C or in PS + 20 mM CaCl_2_ pH 8 at 22°C for 6 h.

Samples were then serially diluted, and phage titers were determined using the spot plating technique.

### Stability of phages under flow and pressure conditions

Phage lysates were processed with a Sartorius Hollow Fiber (HF) 115 cm^2^ cut-off 750 kDA cartridge with a pressure lower than 0.68 Ba at RT. Buffer exchange from growth medium to PBS was done by processing material with five diavolumes of the buffer. Next, phage preparations were concentrated using the same HF system with the same pressure force reducing buffer volume from 300 mL to 40 mL. Three sets of samples were collected and titered on the same day: (i) lysates that were used as loading material for HF, (ii) phage preparations after buffer exchange, (iii) phage preparations after concentration. Each sample was titered in technical replicates, the experiment was repeated twice.

### Phage proteins

Bacteriophage T4 head proteins gp23 and gp24 were produced in the *E. coli* expression system and purified as described by Miernikiewicz et al. (36) with modifications.

Amino acid sequences of both proteins were identical to those in the mature T4 phage head—the proteins lacked 65 (gp23) or 10 (gp24) N-terminal amino acids that are cleaved by gp21 protease during capsid assembly. Genes coding for the proteins were cloned using Gateway technology into pDEST15 vector, allowing for the expression of recombinant products with the N-terminal glutathione *S*-transferase (GST) affinity tag, and expressed in *E. coli* B834(DE3) F^−^ *ompT hsdS*_B_(r_B_^−^, m_B_^−^) *gal dcm met* (DE3) (Novagen). Bacteria were grown in Luria-Bertani broth (LB) high salt (10 g/L of NaCl) culture medium (Sigma-Aldrich) supplemented with appropriate antibiotics (ampicillin and chloramphenicol or ampicillin alone in the case of gp23 or gp24, respectively) at 37°C until OD_600_ reached 0.8–1.0. To facilitate proper folding of gp23, the protein was coexpressed with T4 phage gp31 chaperone, which is a specific phage cochaperonin functionally replacing GroES in the GroES/GroEL chaperonin complex. The chaperone was expressed from pG31t vector, kindly supplied by Professor Andrey Letarov (Russian Academy of Sciences, Moscow). Expression of gp24 did not require additional chaperones. Expression of the recombinant phage proteins was induced with 0.2 mM isopropyl-β-d-thiogalactopyranoside (IPTG) (Thermo Scientific) and conducted overnight at 25°C. Cultures were then centrifuged for 5 min at 8,000 rpm and the supernatants were removed. Harvested bacteria were suspended in PBS, treated with PMSF (1 mM), and lysis was performed by incubation with lysozyme (0.5 or 1.5 mg/mL for gp24 or gp23, respectively) for 6–7 h on ice and by the freeze-thaw method (−80°C). The suspension was then supplemented with Mg^2+^ (up to 1 mM), DNase (20 µg/mL) and RNase (40 µg/mL), and incubated on ice for 3 h. Fractions were separated by two centrifugations (12,000 rpm, 45 min, 4°C). The soluble fraction was filtered through 0.45 µM PVDF filters and incubated with glutathione sorbent slurry (Glutathione Sepharose 4B, GE Healthcare Life Sciences), washed with PBS, and proteins were released by proteolysis with MobiTev Protease1 (10 U/mL) (MoBiTec GmbH) at 10°C; GST tags remained bound in the resin. LPS removal from all protein preparations was done with EndoTrap HD (Hyglos GmbH). Gel filtration FPLC (fast protein liquid chromatography) on a Superdex 75 10/300 Gl column (GE Healthcare Life Sciences) was applied for final separation, and proteins were dialyzed against PBS and filtered through 0.22 µM PVDF filters (Merck Millipore). Proteins were assessed by SDS-PAGE and concentrations were determined using the Bradford assay (Thermo Scientific). Proteins were also run on native PAGE to confirm oligomers formation.

To obtain gp23 variants with single amino acid substitutions corresponding to bypass-24 mutations in the investigated T4 phage mutants (N381S or N384S), mutations were introduced to the pDEST15 plasmid construct bearing wild-type gp23-coding sequence (36) in a PCR with mutagenizing forward primers 5ʹ-CTTCCCGTAGCGTAGTTAACGTT-3ʹ (gp23-N381S) or 5ʹ- CTTCCCGTAACGTAGTTAGCGTTCTGGCTT-3ʹ (gp23-N384S) and the 5ʹ-phosphorylated reverse primer 5ʹ-CGATAATGAAGTTACCTTCACCACGA-3ʹ. The reaction was carried out as follows: initial denaturation at 98°C – 30 s, 35 cycles of 98°C – 10 s, 64°C – 20 s, 72°C – 4 min, followed by final elongation at 72°C – 10 min; and products were then subjected to ligation with Liga5 ligase (A&A Biotechnology) at RT for 30 min. The resulting ligated plasmids were purified using a Clean-up kit (A&A Biotechnology) and used for the transformation of chemocompetent *E. coli* DH5α cells. Once the correct nucleotide sequence of the plasmids was confirmed by direct sequencing (Genomed S.A., Poland), the plasmids were used along with the pG31t vector construct bearing the gp31 chaperone for the cotransformation of *E. coli* B834, and protein production and purification were carried out as described above for the wild-type gp23.

Immunological purity grade protein preparations used to obtain highly responsive protein-specific plasma samples were produced as optimized by Miernikiewicz et al. (36, 50). Immunization of mice was performed as described by Dąbrowska et al. (51).

### Circular dichroism

The stability of gp24, gp23, and two gp23 bypass variants—gp23-N381S and gp23-N384S—was studied by CD. CD spectra were recorded on a J-1500 spectropolarimeter (Jasco, Japan) equipped with a thermostated cell holder and PM-539 detector. The results were expressed as mean residue ellipticity (θ) in deg cm^2^ dmol^−1^ assuming a mean residue weight of 106.8 Da or 109.9 Da per amino acid residue for gp23 and gp23 mutants or gp24, respectively. Spectra were accumulated three to six times. All values were corrected for solvent contributions (PBS). Data pitch, bandwidth, and digital integration time were 1 nm, 2 nm, and 1 s, respectively, at 100 nm min^−1^. For thermal scans, the protein samples were heated from 25 to 80°C (or 25 to 95°C for gp24) with a heating rate of 1°C min^−1^ controlled by a Jasco programmable Peltier element. CD spectra were recorded at 25°C and 80°C and the dichroic activity at 222 nm was continuously monitored every 1°C. The stability of the proteins was expressed in terms of *T*_m_, the temperature at which the concentration of the protein in its folded state equals that in the unfolded state.

## Data Availability

The datasets supporting the conclusions of this article are available in the Open Science Framework (OSF).

## SUPPLEMENTAL MATERIAL

Supplemental materials spectrum.00115-23-s0001.pdf Figures S1 to S4 and Tables S1 to S3 and additional experimental details.

ASM does not own the copyrights to Supplemental Material that may be linked to, or accessed through, an article. The authors have granted ASM a non-exclusive, world-wide license to publish the Supplemental Material files. Please contact the corresponding author directly for reuse.
