# Supplementary material for: Evolution of the T4 phage virion is driven by selection pressure from non-bacterial factors
Source: Microbiol Spectr. 2023 Sep 19;11(5):e00115-23. doi: 10.1128/spectrum.00115-23 (PMC10580926; doi:10.1128/spectrum.00115-23)
Supplement: Supplemental materials — Figures S1 to S4 and Tables S1 to S3 and additional experimental details. [file spectrum.00115-23-s0001.pdf]

Supplemental Materials for

# Evolution of the T4 phage virion is driven by selection pressure from non-bacterial factors

Joanna Majewska[#], Paulina Miernikiewicz, Aleksander Szymczak, Zuzanna Kaźmierczak, Tomasz M. Goszczyński, Barbara Owczarek, Izabela Rybicka, Jarosław Ciekot, Krystyna Dąbrowska[#]

[#] Corresponding authors: Joanna Majewska, joanna.majewska@hirszfeld.pl

Krystyna Dąbrowska, dabrowska@hirszfeld.pl

# Supplemental Materials

**Table S1.** Melting temperatures and changes in enthalpy and entropy of recombinant T4 head proteins: major capsid protein gp23 and its two mutants with a single amino acid substitution N381S or N384S, and head vertex protein gp24 assessed by circular dichroism (CD).

| Protein | $T_m$ (°C) | ΔH (J mol$^{-1}$) | ΔS (J mol$^{-1}$ K$^{-1}$) |
|---|---|---|---|
| **gp23** | 66.1 | 539520 | 1590.5 |
| **gp23-N381S** | 64.5 | 665255 | 1970 |
| **gp23-N384S** | 66.6 | 535559 | 1576 |
| **gp24** | >95 | --- | --- |

**Table S2.** The effect of pepsin on bacteriophage T4 and its mutants. Purified phage preparations ($N$ = 6) ($10^6$ pfu/mL) were incubated in physiological saline (PS) pH 4 or PS pH 4 with pepsin (1 mg/mL) at 37°C or in PS pH 4 at 22°C for 2 h. Phage titers were determined using spot plating. Titer decrease was calculated for the pepsin-treated group with respect to the two non-treated groups.

| **Bacteriophage** | **Phage titer** | | | **Titer decrease [%]** | |
|---|---|---|---|---|---|
| | **A**<br>pH 4,<br>22 °C | **B**<br>pH 4,<br>37 °C | **C**<br>pH 4,<br>pepsin (1mg/mL),<br>37 °C | **(C/A)** | **(C/B)** |
| T4 | 7.17E + 05<br>(± 1.37E + 05) | 6.30E + 05<br>(± 8.83E + 04) | 6.53E + 05<br>(± 1.69E + 05) | 9% | -4% |
| T4ΔHoc | 8.20E + 05<br>(± 8.67E + 04) | 6.13E + 05<br>(± 1.20E + 05) | 7.53E + 05<br>(± 1.28E + 05) | 8% | -23% |
| T4ΔSoc | 1.26E + 06<br>(± 2.42E + 05) | 8.70E + 05<br>(± 1.40E + 05) | 8.80E + 05<br>(± 1.54E + 05) | 30% | -1% |
| T4Δ24byp24_1 | 1.09E + 06<br>(± 1.12E + 05) | 3.12E + 05<br>(± 4.24E + 04) | 1.57E + 05<br>(± 2.11E + 04) | 86% | 50% |
| T4Δ24byp24_2 | 9.50E + 05<br>(± 3.73E + 05) | 3.36E + 05<br>(± 3.40E + 04) | 2.09E + 05<br>(± 2.50E + 04) | 78% | 38% |
| T4ΔHocΔSoc | 8.70E + 05<br>(± 1.92E + 05) | 5.97E + 05<br>(± 8.14E + 04) | 7.00E + 05<br>(± 8.29E + 04) | 20% | -17% |
| T4ΔHocΔ24byp24 | 7.10E + 05<br>(± 1.20E + 05) | 1.18E + 05<br>(± 2.92E + 04) | 5.33E + 04<br>(± 1.65E + 04) | 92% | 55% |
| T4ΔSocΔ24byp24 | 8.37E + 05<br>(± 6.74E + 04) | 1.93E + 04<br>(± 2.24E + 03) | 8.27E + 03<br>(± 1.76E + 03) | 99% | 57% |
| T4ΔHocΔSocΔ24byp24 | 9.57E + 05<br>(± 1.1E + 05) | 3.00E + 04<br>(± 1.35E + 03) | 1.04E + 04<br>(± 2.60E + 03) | 99% | 65% |

**Table S3.** Stability of bacteriophage T4 and its mutants under flow and pressure conditions in the process of purification and concentration of phage preparations using the Hollow Fiber system. Phage lysates were subjected to buffer exchange to PBS and subsequent reduction of buffer volume. Samples were collected throughout the process and phage recovery was calculated. The results of two independent experiments are presented.

| Bacteriophage | | Total number of phage particles | | Total phage recovery [%] |
|---|---|---|---|---|
| | | Lysate | Purified phage preparation | |
| T4 | 1 | 5.85E + 13 | 4.30E + 13 | 73.5% |
| | 2 | 5.25E + 13 | 4.20E + 13 | 80.0% |
| | **Average** | | | **76.8%** |
| T4ΔHoc | 1 | 6.93E + 13 | 4.10E + 13 | 59.2% |
| | 2 | 5.04E + 13 | 4.00E + 13 | 79.4% |
| | **Average** | | | **69.3%** |
| T4ΔSoc | 1 | 5.55E + 13 | 2.90E + 13 | 52.3% |
| | 2 | 3.57E + 13 | 2.10E + 13 | 58.8% |
| | **Average** | | | **55.5%** |
| T4Δ24byp24_1 | 1 | 4.11E + 13 | 3.00E + 13 | 73.0% |
| | 2 | 3.69E + 13 | 2.20E + 13 | 59.6% |
| | **Average** | | | **66.3%** |
| T4ΔHocΔSoc | 1 | 6.75E + 13 | 5.30E + 13 | 78.5% |
| | 2 | 4.08E + 13 | 3.30E + 13 | 80.9% |
| | **Average** | | | **79.7%** |
| T4ΔHocΔ24byp24 | 1 | 2.13E + 13 | 7.60E + 11 | 3.6% |
| | 2 | 4.02E + 13 | 4.30E + 12 | 10.7% |
| | **Average** | | | **7.1%** |
| T4ΔSocΔ24byp24 | 1 | 3.18E + 13 | 2.40E + 13 | 75.5% |
| | 2 | 1.41E + 13 | 1.35E + 13 | 95.7% |
| | **Average** | | | **85.6%** |
| T4ΔHocΔSocΔ24byp24 | 1 | 1.08E + 13 | 5.25E + 11 | 4.9% |
| | 2 | 1.11E + 13 | 7.20E + 11 | 6.5% |
| | **Average** | | | **5.7%** |

**PCR-based method for the detection of nonsense mutations introduced to *soc* gene and identification of mutants in the overall phage progeny**

With Soc being a nonessential protein, identification of mutants bearing nonsense mutations within the *soc* gene in the overall phage progeny following the recombination culture could not be based on differential propagation on nonsense suppressor and wild-type host strains. Therefore, a PCR-based method was applied to differentiate between wild-type and mutated *soc*. Single plaques were isolated and each isolate was used as a template for parallel PCR reactions with a set of primers specific for either the wild-type or modified *soc* region. Exemplary results of such screening are shown in Fig. S1.

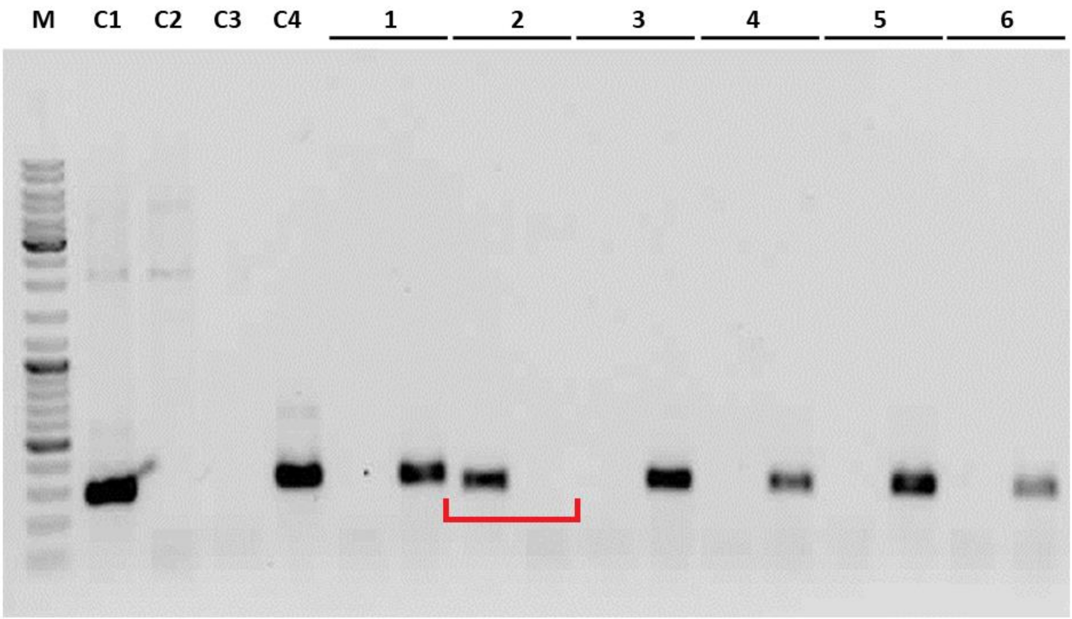


| | | | | | | | | | | | | | | | | |
|---|---|---|---|---|---|---|---|---|---|---|---|---|---|---|---|---|
| pMA + soc_mut | + | + | - | - | - | - | - | - | - | - | - | - | - | - | - | - |
| T4 genome_wt | - | - | + | + | - | - | - | - | - | - | - | - | - | - | - | - |
| Primer_mut | + | - | + | - | + | - | + | - | + | - | + | - | + | - | + | - |
| Primer_wt | - | + | - | + | - | + | - | + | - | + | - | + | - | + | - | + |

**Fig S1.** Identification of Soc-deficient mutants by PCR with two sets of primers specific to wild-type or modified *soc* visualized by electrophoresis. M – DNA mass marker; C1-C4 – controls; 1-6 – isolates. Band profile identifies isolate 2 as a Soc-deficient phage (red). Approximately 800 clones were tested in total; exemplary agarose gel including one positive sample is presented.

**Morphology assessment using transmission electron microscopy (TEM)**

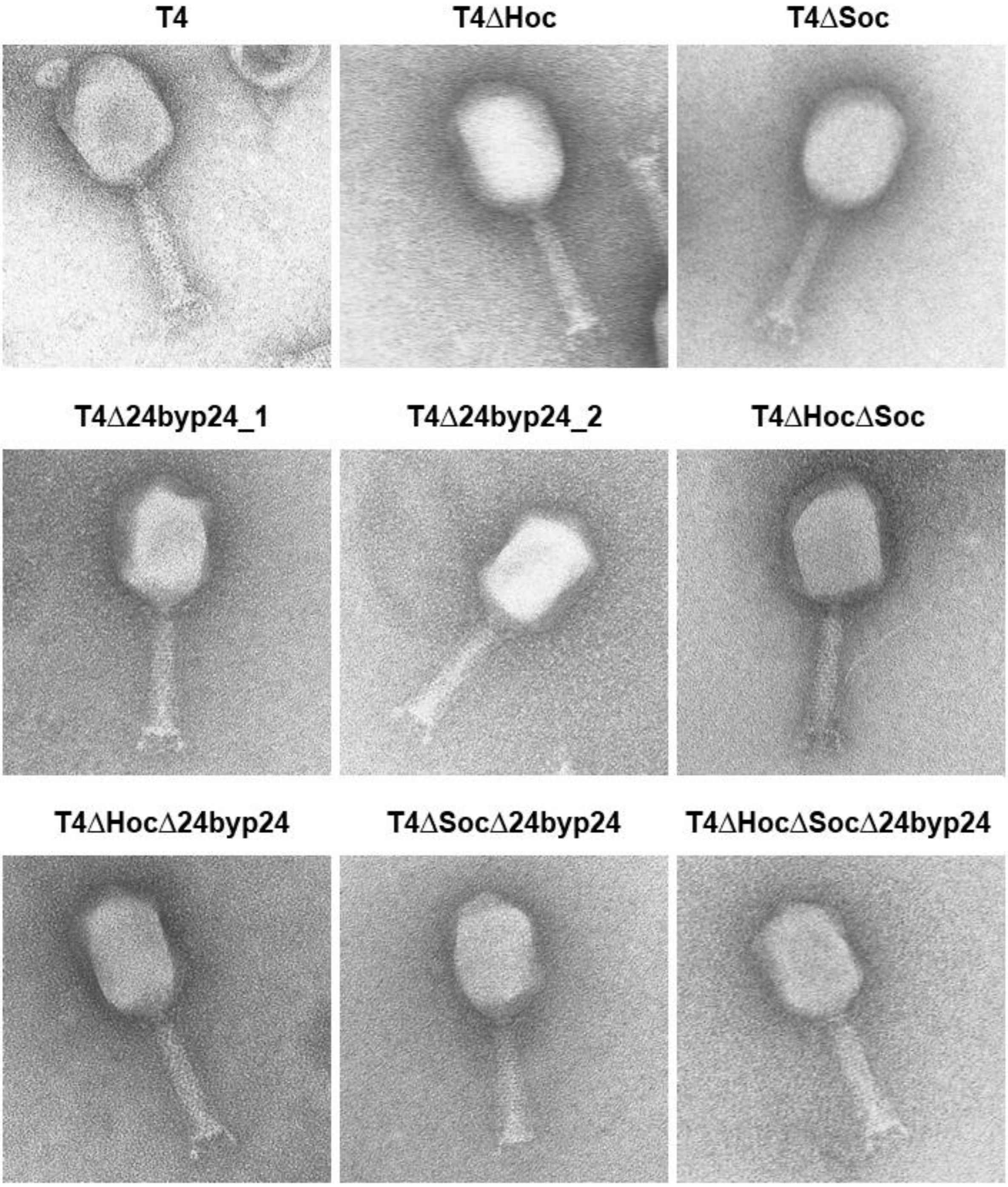


**Fig S2.** Bacteriophage T4 and its mutants visualized under a transmission electron microscope. Phage morphology is consistent with previous reports; Fokine et al. (1) observed in cryo-electron microscopy that electron micrographs of a gp24byp mutant phage contained only particles with a normal head length.

**Protein composition of the head verified in ELISA**

Anti-gp23, anti-gp24, anti-Hoc and anti-Soc protein-specific reference plasma samples collected from mice immunized with these proteins were used in ELISA to verify the capsid composition of the mutants and confirm that the mutations introduced to phage genomes indeed conferred the desired phenotypes. Albumin-specific plasma served as a negative control.

ELISA results (Fig. S3) confirmed that the head protein composition of each phage was correct: wild-type bacteriophage T4 was shown to have all four head proteins, i.e., gp23, gp24, Hoc, and Soc, while the mutants lacked one, two, or three of them. All phages tested positive for the presence of major capsid protein gp23, which cannot be eliminated from the capsid. Presence of bypass-24 mutations in gene *23* confirmed in the genomes of gp24-deficient mutants and accompanying changes in the amino acid sequence of the protein did not interfere with gp23 recognition by the gp23-specific polyclonal antibodies in the reference plasma.

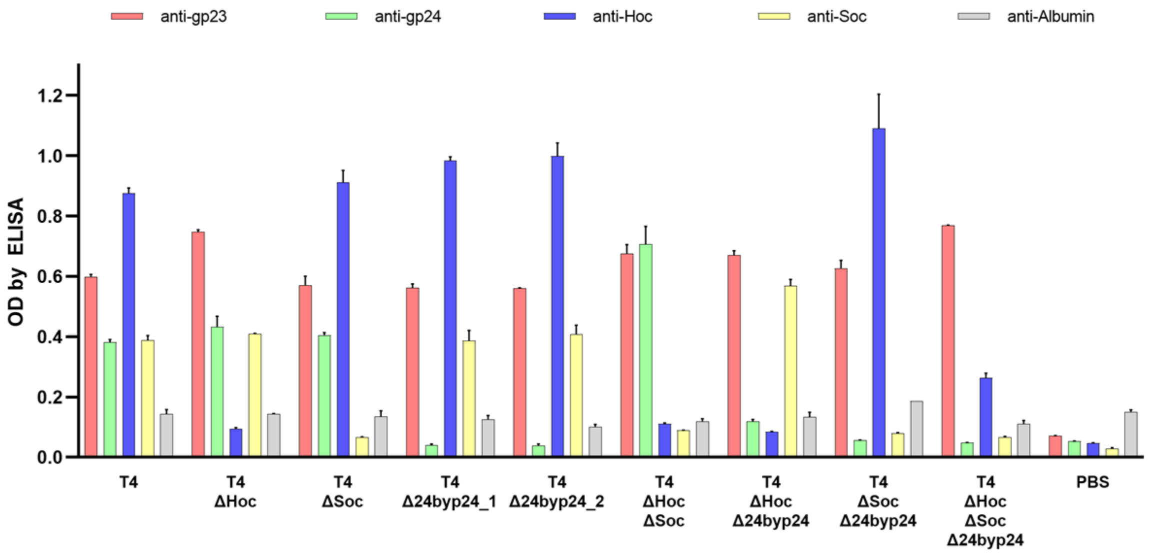


| | | | | | | | | | |
|---|---|---|---|---|---|---|---|---|---|
| gp23 | + | + | + | + | + | + | + | + | + |
| gp24 | + | + | + | - | - | + | - | - | - |
| Hoc | + | - | + | + | + | - | - | + | - |
| Soc | + | + | - | + | + | - | + | - | - |

**Fig S3.** Protein composition of the capsid head of T4 phage and its mutants verified in ELISA with protein-specific plasma samples. Plates were coated with bacteriophage preparations, $5\times10^9$ pfu/mL (in the case of Soc protein, phage preparations were incubated at 80°C for 30 min prior to coating), and the presence of gp23, gp24, Hoc, and Soc in the virions was detected with protein-specific plasma samples obtained from mice immunized with these proteins. Anti-albumin plasma and PBS-coated wells served as negative controls to exclude non-specific interactions.

**Presence of calcium ions improves phage stability when incubated at 37°C**

Detrimental effect of incubation at the temperature of 37°C on gp24- and Soc-deficient phages (Fig. 2) can be counteracted by the introduction of 20 mM $Ca^{2+}$ ions to the phage's microenvironment (Fig. S4). This protective effect of $Ca^{2+}$ ions is, however, also applicable to the wild-type T4; thus it remains unclear whether $Ca^{2+}$ has any specific effect on the mutants or protection is rather a general feature of T4 phage. The effect was most noticeable after at least 24 hours of phage exposure (Fig. S4). Physiological saline (0.9% NaCl solution), which was used as the primary environment in this experiment, has a molar concentration of ~150 mM. Such concentration of sodium ions—well above the ionic strength threshold of 20 mM—had been previously indicated by Szermer-Olearnik et al. (2) as a high-ionic strength environment, preventing clustering of phage particles into aggregates. Moreover, aggregates formed under lower-ionic strength were demonstrated to rapidly destabilize and disperse into single phage virions once ionic strength was increased to 150 mM NaCl. Therefore, the differences in phage titers presented in Fig. S4 are unlikely to result from the further increase of ionic strength caused by the addition of calcium ions to the solution.

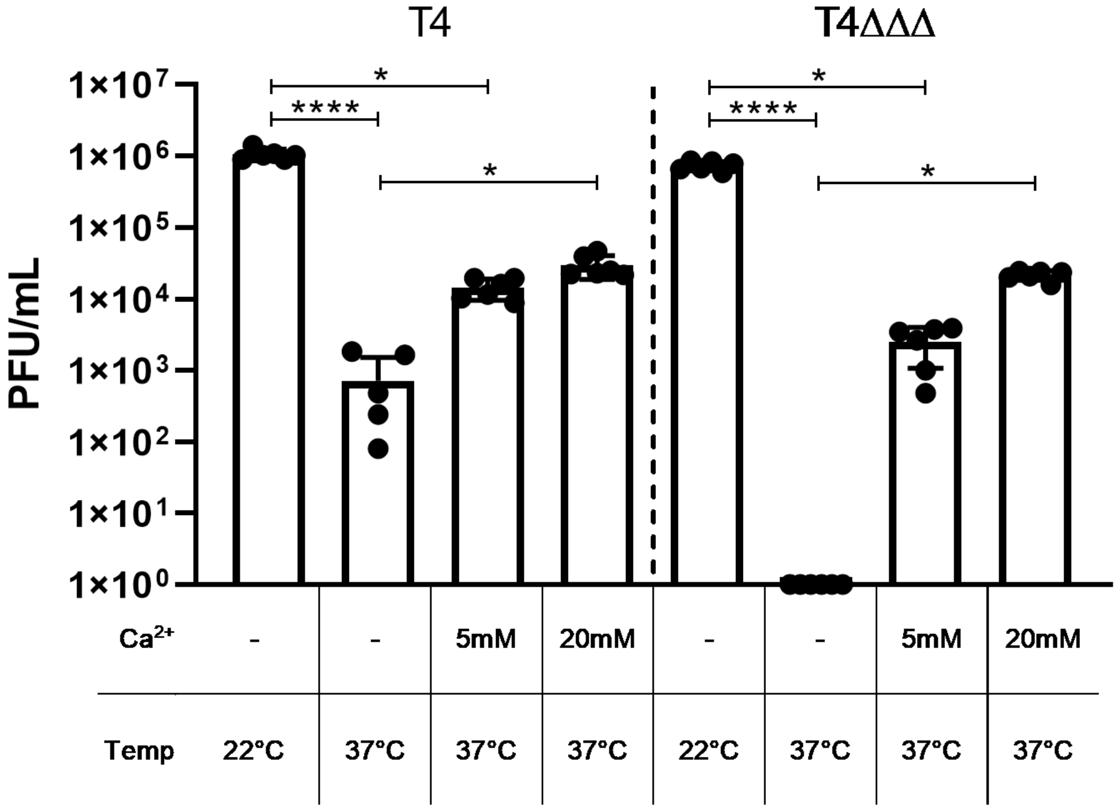


**Fig S4.** The effect of $Ca^{2+}$ ions on the temperature sensitivity of T4 phage and its mutant T4ΔHocΔSocΔ24byp24 (T4ΔΔΔ). Phages (*N* = 6) ($10^6$ pfu/mL) were incubated in PS at 22°C (control) or 37°C or in PS supplemented with $CaCl_2$ (5 mM or 20 mM) at 37°C for 24 h. Phage titers were determined using spot plating. **P* < 0.02; *****P* < 0.0001 (Kruskal-Wallis's *t* test, Dunn's multiple comparisons test).

**References**

1. Fokine A, Battisti AJ, Kostyuchenko VA, Black LW, Rossmann MG. 2006. Cryo-EM structure of a bacteriophage T4 gp24 bypass mutant: the evolution of pentameric vertex proteins in icosahedral viruses. J Struct Biol 154:255–259. https://doi.org/10.1016/j.jsb.2006.01.008

2. Szermer-Olearnik B, Drab M, Mąkosa M, Zembala M, Barbasz J, Dąbrowska K, Boratyński J. 2017. Aggregation/dispersion transitions of T4 phage triggered by environmental ion availability. J Nanobiotechnology 15:32. https://doi.org/10.1186/s12951-017-0266-5
